# Prolactin deficiency in the context of other pituitary hormone abnormalities: Special issue: hypoprolactinemia: a neglected endocrine disorder

**DOI:** 10.1007/s11154-024-09902-z

**Published:** 2024-10-02

**Authors:** Ilan Shimon

**Affiliations:** 1 Institute of Endocrinology, Rabin Medical Center– Beilinson Hospital, 39 Jabotinksi St, Petach Tikva, 4941492 Israel; 2 Faculty of Medical and Health Sciences, Tel Aviv University, Tel Aviv, Israel

**Keywords:** Hypopituitarism; IGF-1, Prolactin, Prolactin deficiency, Lactation, Pituitary failure

## Abstract

Prolactin deficiency is rare. It generally occurs when pituitary disorders, such as large pituitary tumors, pituitary apoplexy, and other conditions associated with sellar mass effect lead to global failure of pituitary function and hypopituitarism. In these situiations, prolactin is commonly the last pituitary hormone affected, after growth hormone and gonadotropins are lost and thyroid-stimulating hormone and adrenocorticotopic hormone secretion is impaired. Prolactin deficiency accompanies several congenital syndromes due to mutations in *PROP1* and *Pit1/ POU1F* and in X-linked IGSF1 deficiency syndrome, and several aqcuired conditions including Sheehan syndrome, IgG4-related hypophysitis, and immune checkpoint-inhibitor-induced hypophysitis. In women, prolactin deficiency prevents lactation following childbirth among other symptoms associated with hypopituitarism. Human prolactin is not available commercially as replacement therapy. However, recombinant human prolactin administered daily to women with hypoprolactinemia and alactogenesis was found to lead to the production of significant milk volume sufficient for lactation.

## Introduction

Prolactin is a 199-amino acid single peptide produced and released by lactotroph cells in the anterior pituitary gland. It belongs to the cytokine class-1 receptor superfamily of peptides and has significant structural homology to placental lactogen and growth hormone (GH) [1]. Prolactin is regulated by tonic inhibition by dopamine which is released from dopamine-producing neurons in the arcuate nucleus of the hypothalamus, crosses the hypothalamus-pituitary portal venous system to the anterior pituitary, and attaches to receptors on lactotroph cells. Circulating prolactin has a broad variety of target tissues, including the mammary gland, prostate, ovaries, cells of the immune system, adipocytes, and liver [2]. It activates target cells by interacting with a pair of single-transmembrane domain type 1 cytokine receptors located on the cell membrane. Intracellular signaling of prolactin involves mainly the JAK/STAT pathway [3].

Clinically, the main physiologic roles of prolactin, recognized since its isolation in the 1970s, involve the mammary gland during pregnancy and following delivery, namely, development and growth of the mammary gland (mammogenesis), milk production (lactogenesis), and continuous milk secretion (galactopoiesis) [2]. Hypoprolactinemia is defined as serum prolactin measured below the lower range for normal, 3–5 ng/ml for women and men by most commercial assays. Accordingly, hypoprolactinemia in women may lead to an inability to produce milk and failure of lactation following delivery (alactogenesis). In addition, hypoprolactinemia in women may impair ovarian function [4]. In men, it has been associated with erectile dysfunction and premature ejaculation, impaired semen parameters, and male infertility [5]. Low prolactin in men has also been correlated with a poor metabolic profile, higher body mass index, diabetes mellitus, and dyslipidemia [6]. Prolactin also has cytokine-like activity [7], contributing to the modulation of T and B cell functions and mediating several anti-inflammatory effects. Therefore, a deficiency of prolactin may adversely affect immune system function.

## Hypoprolactinemia

Prolactin is one of six hormones produced and secreted by five types of cells situated in the anterior pituitary [8] (Table 1). The prolactin-producing lactotrophs represent 15–30% of the hormone-producing cells, depending on the age, sex, and physiologic status of the individual. They are located in the lower lateral region of the pituitary and also randomly spread throughout the gland. Somatotrophs, which produce GH, are located mainly in the anterior wings of the gland and are the most abundant, accounting for 50% of the secretory cells. The remainder include corticotrophs (about 10%), which secrete adrenocortiotropic hormone (ACTH) and are mainly clustered in the center of the gland but also present in the lateral wings; gonadotrophs (up to 10%), which secrete luteinizing hormone (LH) and follicle-stimulating hormone (FSH) and are distrubuted throughout the pars distalis; and thyrotrophs (about 5%), which secrete thyroid-stimulating hormone (TSH) and are situated mainly in the anteromedial portion of the gland.

**Table 1:** Hormone-secreting cells in the anterior pituitary

Cell type	Percent of functioning cells	Location/distribution
Somatotrophs	50	Mainly in the anterior wings of the gland
Lactotrophs	15–30	ln the lower lateral part of the pituitary and also randomly spread throughout the gland
Gonadotrophs	10	Distributed throughout the pars distalis
Thyrotrophs	5	Mostly in the anteromedial part of the pituitary
Corticotrophs	10	Mainly in the center of the gland

Prolactin deficiency in humans is rare. It may be an isolated phenomenon but usually occurs in the context of multiple pituitary hormone deficiencies due to congenital or acquired etiologies or in the setting of structural disorders of the pituitary (Table 2). In the presence of a major insult to the gland, there is a sequential loss of hormones which depends on the distribution, number, location, and potency of the secretory cells. As such, the lactotroph cell population is typically the last to be affected.

**Table 2:** Hypoprolactinemia in congenital and acquired etiologies and in the context of other pituitary deficiencies

Etiology [Ref]	Congenital/ Acquired	Isolated PRL deficiency/combined deficiency	Reversibility
Prolactin gene mutation [15]	Congenital	Isolated PRL	No
Autoabtibodies to lactotrophs [24]	Acquired	Isolated PRL	Unknown
PROP1 gene mutations [9, 10]	Congenital	GH, PRL, TSH, gonadotropins *±* ACTH	No
Pit1/ POU1F1 mutations [10, 11]	Congenital	GH, PRL, TSH	No
IGSF1 gene mutation [12]	Congenital	TSH, PRL, GH excess	No
LHX3 and LHX4 mutations [13, 14]	Congenital	GH, gonadotropin, TSH *±* PRL	No
Sheehan syndrome [16, 17]	Acquired	GH & PRL mainly	No
IgG4 hypophysitis [19, 20]	Acquired	Gonadotropin, ACTH, TSH, GH, PRL (42%)	Unknown
Checkpoint inhibitor hypophysitis [22, 23]	Acquired	ACTH (85%), TSH, gonadotropin, PRL (60%)	Yes
Structural disorders-macroadenomas, craniophayngiomas, pituitary apoplexy, sellar cysts, etc. [25–27]	Acquired	PRL deficiency appears late in the evolution of pituitary failure and hypopituitarism	Rarely

ACTH, adrenocorticotropic hormone; GH, growth hormone; PRL, prolactin;

TSH, thyroid-stimulating hormone

### Congenital etiologies of prolactin deficiency

Pituitary gland development is regulated by pituitary-specific transcription factors, including among others Prophet of PIT-1 (PROP1), PIT-1 (POU1FI), and LHX-3 and LHX-4. Recessive mutations in the *PROP1* gene are associated with GH, TSH, prolactin and are the most common genetic cause of hypopitutarism in humans [9, 10]. Patients with *PROP1* mutations also acquire cortisol deficiency later in life. Magnetic resonance imaging shows a small or normal-sized anterior pituitary.

Pit1 (now called POU1F1) plays a key role in the differentiation and expression of GH, prolactin, and TSH-$\:\beta\:$ genes. It is essential for the development of somatotrophs, lactotrophs, and thyrotrophs in the anterior pituitary. Patients with a *Pit1/ POU1F1* mutation usually present with GH and prolactin deficiencies early in life [10, 11]. TSH deficiency can be highly variable and occurs later in childhood.

The rare mutations in the genes encoding the regulatory transcription factors LHX-3 and LHX-4 are associated with combined deficiencies of pituitary hormones, including prolactin, with variable phenotypes [12, 13].

In addition, human loss-of-function mutations in the immunoglobulin superfamily member 1 (*IGSF1*) gene cause X-linked IGSF1 deficiency syndrome, consisting of central hypothyroidism, hypoprolactinemia, adult macro-orchidism, and excess GH [14].

Recently, familial prolactin deficiency and alactogenesis was reported in a family with a heterozygous stop gain mutation (c.658 C > T) involving a change from CGA to TGA (p.Arg220Ter) in exon 5 of the prolactin gene. This resulted in failure of prolactin production and secretion [15].

### Acquired conditions associated with prolactin deficiency

#### Sheehan syndrome

Sheehan syndrome develops as a consequence of ischemic pituitary necrosis due to massive postpartum hemorrhage [16]. Contributory pathogenic factors include increased pituitary volume and disseminated intravascular coagulation. Lactotroph hyperplasia occurs during pregnancy, increasing pituitary volume and leading to elevated intrasellar pressure. Thereupon, the hypothalamic-pituitary pressure gradient decreases and susceptibility to ischemia increases. Sheehan syndrome is one of the most important causes of hypopituitarism in underdeveloped countries where home deliveries without minimal obstetric support are widespread. Patients may be deficient in a single pituitary hormone or present with panhypopituitarism; the most frequent hormones affected are GH and prolactin. Women experience lack of lactation and failure to resume menstruation after delivery. Some may remain undiagnosed for years following traumatic delivery and therefore do not receive appropriate treatment [17]. Unlike the serial loss of pituitary function commonly encountered with pituitary tumors, in Sheehan syndrome, postpartum necrosis might leave selected functions less affected [14]. The anatomic location of lactotroph cells in the lower lateral region of the adenohypophysis, where they are more vulnerable to destruction by ischemic damage, may explain the preferential loss of prolactin in these women. In patients with a typical obstetric history, the lack of a rise in prolactin following stimulation of thyrotropin-releasing hormone might serve as a sensitive screening test of pituitary damage in postpartum hemorrhage [18].

#### Immunoglobulin G4 (IgG4)-related hypophysitis

IgG4-related hypophysitis is a rare chronic inflammatory condition of the pituitary characterized by elevated serum IgG4 levels and pituitary infiltration by IgG4 plasma cells. The clinical presentation resembles that of other forms of hypophysitis. IgG4-related hypophysitis can lead to anterior hypopituitarism, vasopressin deficiency, and visual impairment [19]. A systematic literature review including a total of 76 patients with IgG4-related hypophysitis reported the following sequence of deficiencies in anterior pituitary hormones: gonadotropins 68.4%, ACTH 63.2%, TSH 59.2%, GH 48.7%, and prolactin 42.1% [20].

#### Immunotherapy-induced hypophysitis

The development of immune checkpoint inhibitors (ICIs) over the last two decades has revolutionized the treatment of solid and hematological malignancies. However, the enhanced immunological activity and autoimmunity triggered by the drugs may cause such endocrine disturbances [21] as thyroid dysfunction, hypophysitis, insulinitis, adrenalitis, and hypoparathyroidism. Immunotherapy-induced hypophysitis occurs in 4–6% of treated patients depending on the type of immunotherapy drug or the combination of agents administered. It is usually diagnosed between 5 and 40 weeks following initiation of ICI treatment. One study reported that among 273 patients with malignant melanoma and prostate cancer treated with ipilimumab, a monoclonal antibody directed against cytotoxic T-lymphocyte antigen-4 (CTLA-4), 9 (3.3%) were diagnosed with hypophysitis. All had corticotropin deficiency. In addition, 7 and 6 patients had deficits in gonadotropin and thyrotropin, respectively, and one patient had GH deficiency; 4 patients had undetectable levels of prolactin [22]. Albarel et al. [23] summarized several longitudinal cohort studies of ICI-induced hypophysitis including 76 patients who received anti-CTLA-4 antibodies, anti-programmed death receptor-1(PD-1) antibodies, antibodies targeting PD-1 ligand (PD-L1), or combination treatments. Corticotropin deficiency was very common, diagnosed in 85% of patients. Thyrotropin and gonadotropin deficiencies were also very frequent at presentation, and low prolactin was reported in 60%. The hormonal deficiencies usually improved, except for corticotroph function that usually remained diminished.

#### Autoimmunity

Isolated prolactin deficiency with puerperal alactogenesis was reported in a woman with autoantibodies that specifically recognized lactotrophs but not prolactin itself or any other pituitary cells or pituitary hormones [24]. She resumed lactation following treatment with recombinant human prolactin, but the alactogenesis returned after treatment was completed.

#### Structural disorders of the pituitary

Structural disorders of the hypothalamic-pituitary area are frequently associated with deficiencies in anterior pituitary hormones including prolactin. They consist of pituitary macroadenomas, craniophayngiomas, pituitary apoplexy, and sellar cysts. Other causes are post-pituitary surgery status and sellar radiotherapy or head trauma. All of these can also lead to posterior pituitary damage with vasopressin deficiency. In a series of 100 consecutive patients with disorders of the hypothalamic-pituitary area, prolactin deficiency was diagnosed in 27, severe (< 3 ng/ml) in 14 and mild (3–5 ng/ml) in 13. Underlyiing disorders included pituitary apoplexy, non-functioning pituitary macroadenoma, and craniopharyngioma. The rate of severe prolactin deficiency was higher (38%) in patients with four additional hormonal deficits (in ACTH, TSH, gonadotropins, and vasopressin) than in patients in whom only one or no other pituitary hormone was affected. Patients with severe prolactin deficiency had more extensive pituitary damage, with destruction of lactotrophs, thyrotrophs, somatotrophs, and corticotrophs [25]. Traumatic brain injury may occasionally cause hypothalamic-pituitary dysfunction, associated with gonadotropins and IGF-1 deficiency as the most common alterations, followed by corticotropin and thyrotropin deficiency. Hyper- and hypoprolactinemia may also occur [26].

Another study of 369 patients with a variety of hypothalamic-pituitary disorders (including 75 nonfunctioning pituitary adenomas, 62 with Cushing’s disease, 23 craniopharyngiomas, 118 post-head radiotherapy, 11 germinomas, 10 gliomas, 5 meningiomas, and 4 pituitary apoplexy, reported that 22 (6%) showed evidence of severe acquired prolactin deficiency (< 2.5 ng/ml) [27]. Surprisingly, 13 of these 22 patients had been previously treated for Cushing’s disease; the remainder had craniopharyngiomas, optic gliomas, nonfunctioning adenomas, and pituitary apoplexy. All prolactin-deficient patients also had severe GH deficiency, gonadotropin deficiency, and central hypothyroidism. Eight patients (36%) were vasopressin-deficient [27]. The authors proposed that in Cushing’s disease, the excess of glucocorticoids may have an inhibitory effect on prolactin release. The findings that none of the patients had isolated prolactin deficiency, and all had evidence of extensive pituitary hormone deficiency, support the late development of prolactin deficiency in the evolution of anterior pituitary hormone failure. Thus, the presence of acquired prolactin deficiency might serve as a reliable marker of severe hypopituitarism.

In a cohort study of 162 adults with severe GH deficiency, prolactin deficiency was independently associated with lower levels of insulin like growth factor 1 (IGF-1) [28]. The authors suspected that the prolactin deficiency in these cases reflected global pituitary damage and a severe degree of GH deficiency associated with low IGF-1 serum levels. Alternatively, they speculated that when GH is absent, prolactin may be involved in IGF-1 generation in the liver, and when GH and prolactin levels are low, IGF-1 concentration may decrease. This hypothesis is based on the structural homology between prolactin and GH, their respective receptors, and their signal transduction pathways [1]. Thus, prolactin deficiency may be a surrogate marker for the severity of anterior pituitary failure in adults, and specifically, of GH/IGF-1 deficiency.

## Replacement treatment

While ACTH and TSH deficiencies always require adequate replacement treatment, sex hormone replacement therapy needs an individual benefit-risk assessment according to the age and gender of the patient and the presence of comorbidities. GH replacement therapy is frequently controversial in the context of a large pituitary remnant following adenoma or caraniopharyngioma resection. Prolactin is the only pituitary hormone not available commercially as replacement therapy.

The biological activity of recombinant human prolactin was studied in 9 healthy premenopausal women who were given daily injections for 7 days. Prolactin levels increased in all cases. Five women experienced expressible galactorrhea [29]. Others treated postpartum mothers who had congenital or acquired prolactin deficiency and failure of milk production with subcutaneous injections of recombinant human prolactin (60 µg/kg) twice daily. Serum prolactin levels rose and milk volume increased significantly [24, 30].

## Summary

An undetectable or very low prolactin level may accompany several pituitary conditions, congenital or acquired. Prolactin deficiency may occur as an isolated phenomenon but generally as part of combined pituitary deficiency and panhypopituitarism. Some conditions are reversible, but most lead to permanent lactotroph damage typically with damage to other pituitary axes as well. Studies in large cohorts of patients with various pituitary pathologies and with mass effects show that hypoprolactinemia mostly reflects global pituitary damage, a severe degree of GH deficiency, low IGF-1 concentration, and impaired production and secretion of three to four other pituitary hormones. Prolactin deficiency frequently appears last in the pattern of development of anterior pituitary failure, with the loss of GH and gonadotropins first, followed by TSH and ACTH.

## Data Availability

No datasets were generated or analysed during the current study.

## Abbreviations

ACTH: Adrenocorticotropic hormone
CTLA4: Cytotoxic T-lymphocyte antigen 4
GH: Growth hormone
ICI: Immune checkpoint inhibitor
IGF-1: Insulin like growth factor 1
TSH: Thyroid-stimulating hormone
